# Manganese‐enhanced magnetic resonance imaging combined with electrophysiology in the evaluation of visual pathway in experimental rat models with monocular blindness

**DOI:** 10.1002/brb3.731

**Published:** 2017-05-22

**Authors:** Zuohua Tang, Jie Wang, Zebin Xiao, Xinghuai Sun, Xiaoyuan Feng, Weijun Tang, Qian Chen, Lingjie Wu, Rong Wang, Yufeng Zhong, Wentao Wang, Jianfeng Luo

**Affiliations:** ^1^ Department of Radiology Eye and ENT Hospital of Shanghai Medical School Fudan University Shanghai China; ^2^ Department of Radiotherapy Eye and ENT Hospital of Shanghai Medical School Fudan University Shanghai China; ^3^ State Key Laboratory of Medical Neurobiology Department of Ophthalmology Eye and ENT Hospital of Shanghai Medical School Institutes of Brain Science Fudan University Shanghai China; ^4^ Department of Radiology Huashan Hospital of Shanghai Medical School Fudan University Shanghai China; ^5^ Department of Otolaryngology Eye and ENT Hospital of Shanghai Medical School Fudan University Shanghai China; ^6^ Central Laboratory Eye and ENT Hospital of Shanghai Medical School Fudan University Shanghai China; ^7^ Health Statistics Shanghai Medical School Fudan University Shanghai China

**Keywords:** auditory evoked visual cortex responses, manganese‐enhanced MRI, monocular blindness, visual evoked potentials, visual pathway

## Abstract

**Purpose:**

Our study aimed to explore the feasibility of manganese‐enhanced magnetic resonance imaging (MEMRI) combined with visual evoked potentials (VEP) and auditory evoked visual cortex responses (AVR) in evaluating for the establishment of visual/auditory compensatory pathways after monocular blindness.

**Materials and Methods:**

A total of 14 healthy neonatal male Sprague‐Dawley rats were randomly divided into two groups (*n *= 7 for Groups A and B). Right optic nerve (ON) transection was performed on the 7 rats of Group A to obtain a monocularly blind model, and the 7 rats of Group B were chosen as the control group. Four months later, 400 mmol MnCl_2_ was injected into the left eye in both groups via intravitreal injection. The changes in the visual pathways projected from the blind eye and the remaining eye in Group A and the normal eyes in Group B were compared to determine if new visual compensatory pathways were established. Additionally, VEP tests were performed to determine complete blindness, and AVR examinations were performed to help identify the generation of auditory compensatory function.

**Results:**

The VEP test indicated complete visual loss after ON transection. In the monocularly blind rats, the contrast‐to‐noise ratio (CNR) of ON, optic tract (OT), lateral geniculate nucleus (LGN), superior colliculus (SC), optic radiation (OR) and visual cortex (VC) of visual pathway projected from the left eye was significantly higher than that of the right pathway (*p *<* *.001). Moreover, the CNR of ON, OT, LGN, SC, OR and VC in the visual pathway projected from the left eye of monocularly blind rats was significantly lower than those of normal rats (*p *<* *.05). The AVR results revealed that the corresponding bilateral visual cortex in monocularly blind rats did not respond to the auditory stimulus or showed dissimilation with the low frequency.

**Conclusion:**

MEMRI combined with electrophysiology, including VEP and AVR, may be potentially helpful in the evaluation of the possible generation of new visual/auditory compensatory pathways after monocular blindness.

## 1 INTRODUCTION

Because of the increasing number of monocularly blind patients with total loss of optic nerve function caused by glaucoma and other diseases (Cumberland & Rahi, 2016; Rizzo et al., 2017), it is important to explore whether the structures of the central visual system have irreversible changes; this information is closely related to the feasibility of artificial vision (Marchini et al., 2016) and gene therapy (Ashtari et al., 2015), as well as the choice of therapeutic target (Cumberland & Rahi, 2016). This subject is also a topic of considerable interest in ophthalmology. To date, many studies have used numerous advanced technologies to investigate the changes in the visual pathway after monocular blindness, such as blood oxygen level‐dependent functional magnetic resonance imaging (BOLD‐fMRI), visual evoked potentials (VEP), horseradish peroxidase (HRP) and other techniques (Chow et al., 2011; Dietrich, Hertrich, & Ackermann, 2015; Jeffery & Thompson, 1986; Mastropasqua et al., 2015; Qu, Dong, Sugioka, & Yamadori, 1996; Toldi, Feher, & Wolff, 1996).

Manganese ion (Mn^2+^) is a paramagnetic contrast agent and a calcium analog for MRI, which prompts its application as a preferred contrast agent to trace the neural pathway (Pautler, Silva, & Koretsky, 1998). Recently, the application of manganese‐enhanced MRI (MEMRI) to the study of visual pathways has increased gradually (Chan, Fu, Hui, So, & Wu, 2008; Chan et al., 2011, 2012; Pautler et al., 1998; Thuen et al., 2005; Watanabe, Michaelis, & Frahm, 2001). Nevertheless, to the best of our knowledge, only one report focusing on the changes in bilateral visual pathways in monocularly blind animals using MEMRI has been published (Chan et al., 2012). In that study, Chan et al. (2012) utilized MEMRI and diffusion tensor imaging (DTI) to assess the retinal and callosal projections in normal neonatal brains and after early postnatal visual impairment. The results revealed a 2–10% signal increase in the ipsilateral superior colliculus of the MnCl_2_‐injected eye in monocularly blind adult rats, normal neonatal rats and normal adult mice. However, the changes of signal intensity in the bilateral optic tracts, optic radiations and visual cortices of monocularly blind models have not been studied in depth.

Thus, our study aimed to evaluate the changes of signal intensity to explore the possible establishment of reorganization in bilaterial visual pathway of monocularly blind rats using MEMRI. Furthermore, the demonstration of MEMRI were compared with the results of VEP and auditory evoked visual cortex responses (AVR) to verify the generation of a visual compensatory pathway. Moreover, because of the possible deviation caused by a large injured area within the orbit after monocular enucleation (Chow et al., 2011; Horton & Hocking, 1998), our study used optic nerve transection instead of monocular enucleation to establish a monocularly blind rat model, which has proven to be an easy and repeatable method to perform in our experimental center. All these attempts are expected to lay a foundation for further research in the anatomical structure, morphology, functional localization and compensation of visual pathways in healthy human beings and in monocularly blind people, which will be helpful to select optimal therapeutic schedules for the blind.

## 2 MATERIALS AND METHODS

This study was conducted in strict accordance with the recommendations in the Guide for the Care and Use of Laboratory Animals of Fudan University and according to local and international ethical guidelines. The protocol was approved by the Committee on the Ethics of Animal Experiments at the Eye & ENT Hospital.

### 2.1 Animals and experimental protocol

**Figure 1:**
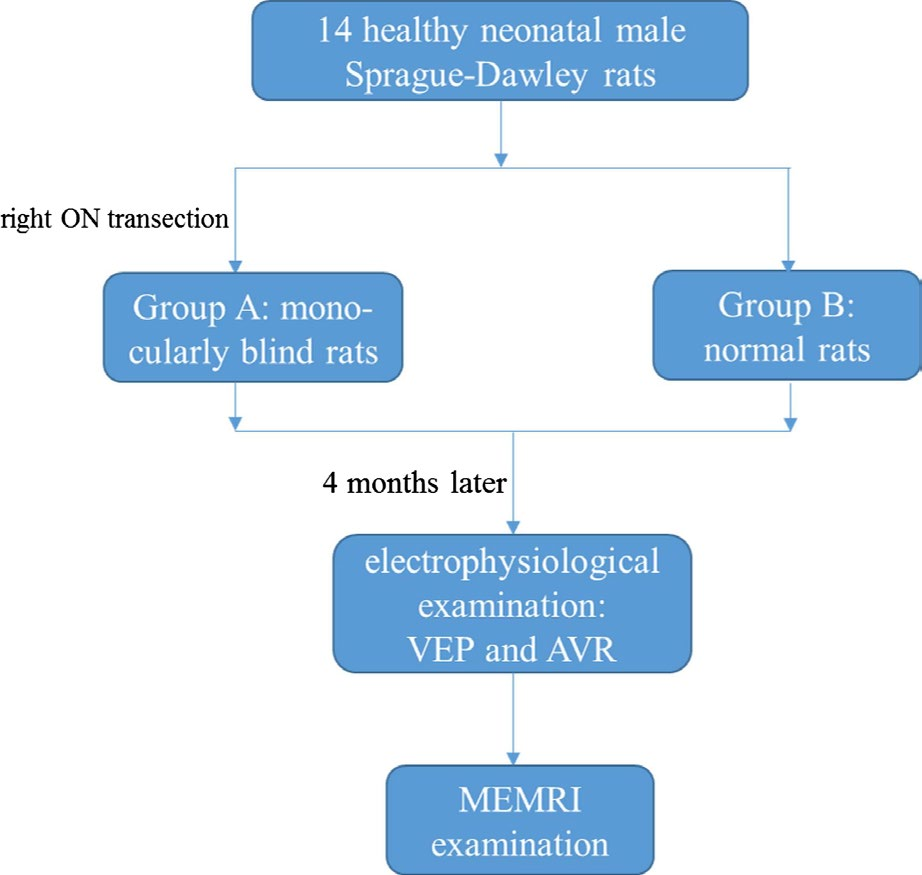
Experimental flow diagram

As shown in Figure 1, a total of 14 healthy neonatal male Sprague‐Dawley rats (RRID:RGD_70508) (1 week after birth, weight 250 ± 30 g, purchased from Experimental Animal Center, Shanghai Medical School, Fudan University) were randomly divided into two groups (*n *= 7 for Groups A and B). No statistical method was used to calculate the sample size in this study. Right optic nerve (ON) transection was performed on the 7 rats of Group A at 3 weeks after birth [the critical period of the establishment of the cross‐model plasticity in rats (Antonini, Fagiolini, & Stryker, 1999)] to obtain a monocularly blind model (right eye), and the 7 rats of Group B were chosen as the control group. We used 0.8 g of solid manganese chloride (MnCl_2_; Trevigen, Shanghai Haoran Biological Technology Co., LTD) dissolved in 10 ml of distilled water, which yielded a final concentration of MnCl_2_ of approximately 0.2 mol/L. Four months later, 400 mmol MnCl_2_ was injected into the left eye in both groups via intravitreal injection without blinding to the group assignments. The changes in the visual pathways projected from the blind eye and the remaining eye in Group A and the normal eyes in Group B were compared to determine if new visual compensatory pathways were established. Additionally, VEP tests were performed to determine complete blindness, and AVR examinations were performed to help identify the generation of auditory compensatory function.

All rats were individually housed and maintained at 25°C with a 12/12 hr reversed light/dark schedule. The duration of fasting and water‐deprivation was approximately 12 hr prior to the experiments. Body temperature was maintained at 38°C. A mixture of ketamine (Romping, 20 mg/ml) and xylazine (Kerala, 50 mg/ml) was administered by intramuscular injection in the posterior superior muscle group of the thigh, and the doses were 0.1 ml (ON transection), 0.4 ml (VEP and AVR), 0.3 ml (intravitreal injection) and 0.4 ml (MRI examination).

### 2.2 Surgical procedures

#### 2.2.1 Animal model establishment for monocular blindness (ON transection)

Optic nerve transection was performed intraorbitally according to procedures that are standard and regular in our institution. Briefly, the right eye was used as the experimental eye. Under anesthesia, a small incision was made in the right upper palpebral conjunctiva, and the right superior oblique muscle was cut. By lifting up this muscle, we stripped it until we can see the root of the ON (which is attached to the sclera) under an operating microscope (YZ20P6; Liuliu Visual Technology Co., Suzhou, China). After the pink dura sheath of the ON was open logitudinally, the white ON was exposed. Then, we transected the ON 0.5 mm away from the sclera and cut a small segment with ophthalmic microscissors. A small section of ON was removed to ensure the complete transection. Caution was taken to avoid injury to the retina artery. After surgery, the eye fundus of each experimental eye was checked to verify the integrity of retinal blood flow. The incised upper and lower conjunctivae were sutured and reset. Tobramycin eye ointment was applied within the conjunctival sac.

#### 2.2.2 VEP test and analysis

**Figure 2:**
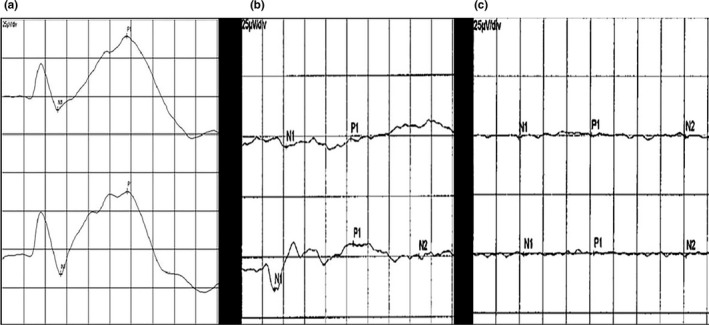
VEP waveforms of normal rats, monocularly blind rats and at baseline. Note: the upper line represents the right eye FLASH VEP response, and the lower line represents the left eye FLASH VEP response. a: normal rats; b: monocularly blind rats; c: background response (baseline VEP)

Visual evoked potentials tests were performed on an electrophysiological diagnostic system (RETI‐PORT21, ROLAND Consult, Brandenburg, Germany). The collecting electrode (pin electrode) which recorded flash VEP was placed at 3 mm forward the tip of lambdoidal suture to receive flash stimuli. The reference electrode was placed at the center of anterior fontanelle. The pin electrode placed on the nose was used as grounding electrode. With the contralateral eye covered by an opaque black eyeshade, flash stimuli from a visual electrophysiological system (FLASH on) were presented to one eye, and the VEP response was recorded from the contralateral visual cortex for three times. The interval value between the positive and negative peak of N1P1 measured by the computer was used to represent the VEP response (Figure 2). The VEP response was repeatedly recorded as the baseline value (background VEP) when the flash stimuli were removed (FLASH off) for three times, which was performed as a comparative study with the VEP response to FLASH on. Then the average results of VEP responses were compared with MEMRI data for the corresponding visual pathway.

#### 2.2.3 AVR detection and analysis

Binaural AVR in both groups were performed on an electrophysiological detector (TDT System 3, Tucker‐Davis Technologies Inc., Alachua, USA). The three electrodes were placed respectively as follows: (1) reference electrode at the top of the head; (2) negative electrode (grounding electrode) in the nasal dorsum; (3) collecting electrode in the front of the occipital tuberosity and 2 mm to the midline. After general anesthesia, the AVR of the left ear was first tested. With an implanted speaker placed in the external auditory canal of the left ear, the threshold values of AVR were detected for four different frequencies, 8000 Hz, 16,000 Hz, 24,000 Hz and 32,000 Hz. For the right ear, we adopted the same method and steps as for the left side and only moved the implanted speaker and the collecting electrode from the left side to the right side. Four sets of data from the AVR were recorded for three times, including left auditory stimulation‐evoked left visual cortex responses (LSLV), left auditory stimulation‐evoked right visual cortex responses (LSRV), right auditory stimulation‐evoked left visual cortex responses (RSLV) and right auditory stimulation‐evoked right visual cortex responses (RSRV). Then the average AVR results were compared with MEMRI data for the corresponding visual pathway.

#### 2.2.4 MnCl_2_ injection into the left eye via intravitreal injection

Anesthetized rats were placed on a sterile drape, and under microscopic observation (YZ20P6; Liuliu Visual Technology co.), a round hole was opened in the acoustic vesicle. A total of 2 μl of 0.2 mol/L MnCl_2_ and a 0.5 μl air bubble were injected into the left eye of rats in each group via intravitreal injection. A 25‐μl microsyringe (MICROLITER^TM^ Syringes, Hamilton Co., USA) was maintained in place for 5 min and slowly extracted 1 min after the injection. After T1W scanning on the MRI, a high MRI signal in the right superior colliculus 24 hr after the administration of MnCl_2_ indicated successful labeling of the visual pathway, while the lack of a high signal indicated failure. There were no subject lost due to poor enhancement in the right superior colliculus in our study.

### 2.3 MRI protocols

MRI scanning was performed using a Siemens–MAGNETOM Verio 3.0 Tesla MR scanner with a gradient of 45 mT/m and a maximum switchover rate of 200 T/m/s. The anesthetized animals were placed in the rat surface coil in a prone position with their heads in the central part of the coil. Thin sheeting was used to keep the animals warm, and tape was used to fix the chest to moderately restrict respiratory movement.

T1‐weighted axial images and T1‐weighted three‐dimensional fast low angle shot (3D FLASH) sagittal images covering the entire visual pathway were scanned using a gradient echo sequence. T1‐weighted imaging was performed with TE/TR = 13 ms/400 ms, FOV = 78 mm × 78 mm, slice thickness = 1.5 mm, slice gap = 0 mm, matrix size = 256 × 256, number of excitations = 2, flip angle = 90°, and number of slices = 8. T1‐weighted 3D FLASH imaging was performed with TE/TR = 4.3 ms/12 ms, slice thickness = 0.2 mm, FOV = 78 mm × 78 mm, matrix size = 384 × 384, number of excitations = 12, flip angle = 25°, and number of slices = 112. The total scan time was approximately 40 min.

### 2.4 MRI Data analysis

**Figure 3:**
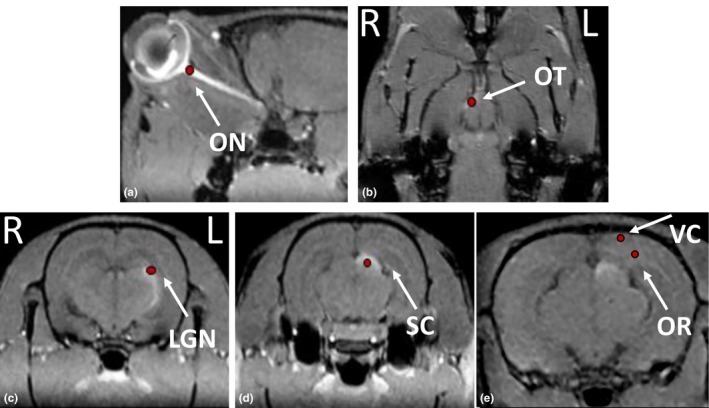
Representative manganese‐enhanced MR images to display the placement of regions of interest for the measurement of signal intensities. a, the level of optic nerve; b, the level of optic tract; c, the level of lateral geniculate nucleus; d, the level of superior colliculus; e, the level of optic radiation and visual cortex

Magnetic resonance image data analysis was performed at a Siemens MR post‐processing workstation (Verio; Siemens Healthcare, Erlangen, Germany). Multiplanar reformation (MPR), maximum intensity projection (MIP) and thin‐slab maximum intensity projection (TSMIP) were performed to reconstruct the entire bilateral visual pathways by the data of 3D FLASH T1WI. The anatomical locations with enhanced contrast were identified with respect to neuroanatomical landmarks of the skull and the brain on post‐contrast MR images. The signal intensity (SI) of various anatomical structures was detected on non‐enhanced and Mn^2+^‐enhanced T1WI, respectively. As demonstrated in Figure 3, the SI of the visual pathways was obtained using a circle‐type region of interest (ROI) located in the center of six different regions based on the resolved anatomical structures and the rat brain atlas, including the left ON, right optic tract (OT), right lateral geniculate nucleus (LGN), right superior colliculus (SC), right optic radiation (OR) and right visual cortex (VC), with the size of the ROI being approximately 2–3 mm^2^. Importantly, the ROI placement of OR was defined as previously reported by Dai, Wang, Shan, Niu, & Lei (2017). All the positions where the ROIs were placed were verified independently by two neuroanatomists blinded to the group assignments. Additionally, the same size and type of ROI was placed at the same anatomical structures in normal rats, as well as in the air. The average SI and standard deviation (SD) of the ROI could be directly acquired using in‐house software analysis of MR.

Based on the enhancement peak time of the visual pathway, some images of peak time points were chosen for a comparative analysis with the contralateral images and the corresponding images from normal rats. The contrast‐to‐noise ratio (CNR) was defined as CNR = 0.655·(S_Mn_‐S_0_)/SD_air_ (Firbank, Coulthard, Harrison, & Williams, 1999). This index, which indicates the enhancement extent of bilateral visual pathways, represents the relative ratio of enhancement extent between the images with MnCl_2_ administration in the left eye and the corresponding images of normal rats with no MnCl_2_ injection. In the formula, S_Mn_ and S_0_ represent the SI value of the ROI within the aforementioned six regions after and before MnCl_2_ administration, respectively, and SD_air_ is twice the average SD value of the ROI of air.

### 2.5 Statistical analysis

All data were expressed as mean ± SD because of their normal distribution and equal variance which were tested by the Kolmogorov‐Smirnov test for normality and the Levene test for variance homogeneity. The information about the group assignments were anonymised during the analysis. Statistical analysis was performed with Statistical Package for the Social Sciences (RRID:SCR_002865) (SPSS, Version 18.0, Chicago, IL, USA). The independent samples *t*‐test was adopted to compare the difference in VEP before and after monocular deprivation, the difference in VEP after monocular deprivation and baseline VEP, and the difference in bilateral AVR between normal rats and monocularly blind rats. One‐way ANOVA and post‐hoc Bonferroni's multiple comparison test were used to compare the difference in the CNR between the bilateral visual pathway in monocularly blind rats, as well as between monocularly blind rats and normal rats in visual pathway projected from the right eye. The level of significance was set at *p *<* *.05.

## 3 RESULTS

**Figure 4:**
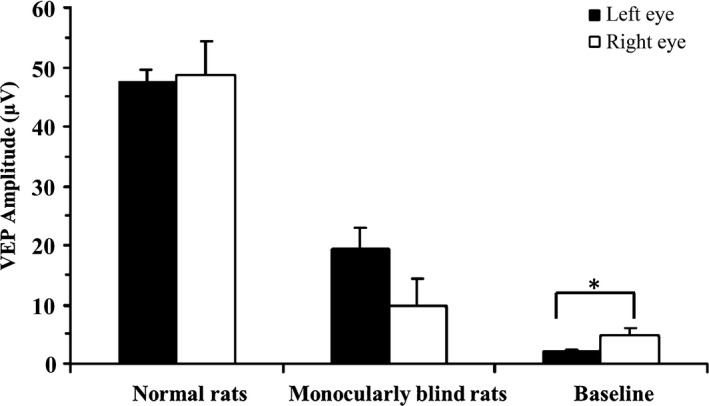
Comparison of VEP before and after monocular blindness and at baseline between the left and right eyes. Baseline, visual cortex of spontaneous activity without stimulation conditions; VEP, visual evoked potential. *: *p *<* *.05

**Figure 5:**
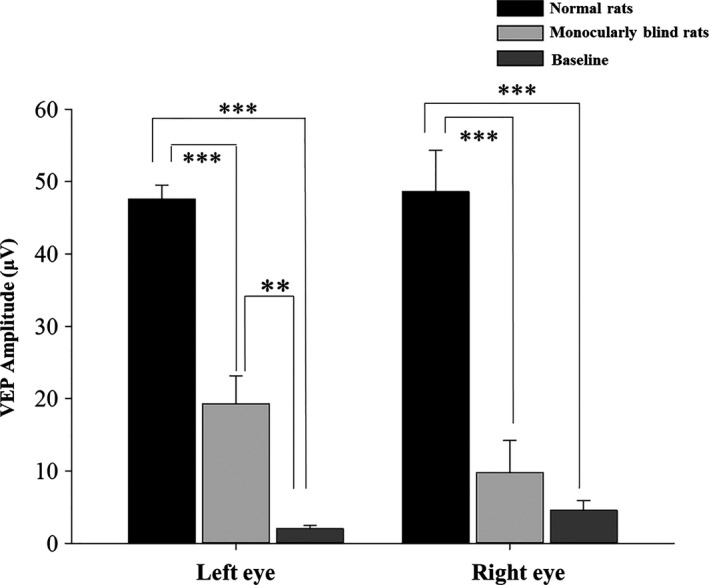
Comparison of VEP in both eyes before and after monocular blindness, and comparison of VEP after monocular blindness and at baseline. Baseline, visual cortex of spontaneous activity without stimulation conditions; VEP, visual evoked potential. ***: *p *<* *.001; **: *p *<* *.01

In the monocularly blind rats, there was no significant difference in the N1P1 amplitude of the VEP between the bilateral visual pathways (*p *=* *.135), even after the removal of background amplitude (*p *=* *.065). Additionally, the bilateral VEP of rats after the right ON transection decreased significantly compared with those before monocular deprivation (*p *<* *.001). In particular, the VEP of the transection side decreased significantly and was nearly equal to the baseline VEP, with no significant difference between them (*p *=* *.286) (Tables 1, 2 and Figures 4, 5).

**Table 1:** Comparison of VEPs (before and after monocular blindness and baseline) between bilateral eyes

	Stimulate the left eye	Stimulate the right eye	*p* value
Before monocular blindness	47.6 ± 1.9	48.6 ± 5.7	.870
After monocular blindness	19.3 ± 3.8	9.8 ± 4.4	.135
Baseline	2.0 ± 0.5	4.6 ± 1.3	.101
After‐Baseline	17.2 ± 4.0	5.1 ± 2.9	.065

Baseline, visual cortex of spontaneous activity without stimulation conditions; After‐Baseline, an absolute value, indicating spontaneous reaction of the VEP signal after removing the Baseline.

**Table 2:** Comparison of VEP between before and after monocular blindness, after monocular blindness and baseline, respectively

*p* value	BAMB	AMBB
Stimulate the left eye	.00006***	.00113**
Stimulate the right eye	.00031***	.28607

BAMB, comparison of VEP between before and after monocular blindness; AMBB, comparison of VEP between after monocular blindness and baseline; Basement, visual cortex of spontaneous activity without stimulation conditions.

***p <* *.001, **p <* *.01.

**Figure 6:**
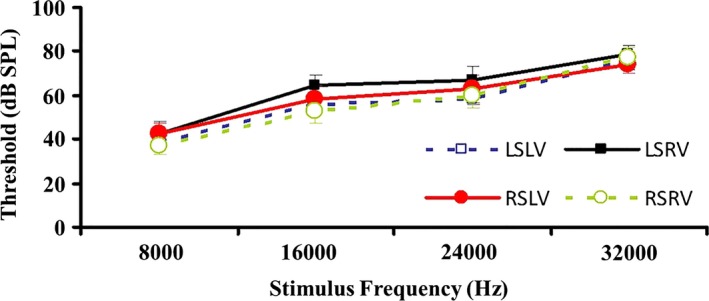
Comparison of AVR parameters in rats after right optic nerve transaction. AVR, auditory evoked visual cortex responses

**Figure 7:**
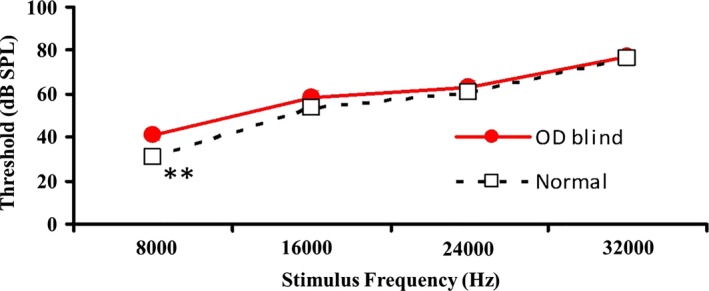
Comparison of bilateral AVR between monocular blind and normal rats at different frequencies. AVR, auditory evoked visual cortex responses. **: *p *<* *.01

As shown in Table 3 and Figure 6, the four curves for LSLV, LSRV, RSLV and RSRV were generally similar. A significant difference existed at the low frequency (*p *=* *.002), but we found no significant difference at the other three frequencies (*p *>* *.05) (Table 4 and Figure 7).

**Table 3:** Comparison of bilateral AVR in monocularly blind rats

	Frequency (Hz)			
	8000	16,000	24,000	32,000
Left				
LSLV	39 ± 5	56 ± 5	59 ± 7	76 ± 6
LSRV	43 ± 5	64 ± 5	67 ± 6	79 ± 4
Right				
RSLV	43 ± 5	59 ± 6	63 ± 6	74 ± 4
RSRV	37 ± 4	53 ± 6	60 ± 6	77 ± 5
*p* value				
LSLV vs. LSRV	.4072	.1996	.0167	.3559
LSLV vs. RSLV	.4481	.5222	.5338	.7663
LSLV vs. RSRV	.6891	.6314	.8461	.7882
LSRV vs. RSLV	1.0000	.3559	.5546	.3559
LSRV vs. RSRV	.2797	.0300^a^	.3341	.7663
RSLV vs. RSRV	.0300^a^	.1030	.1723	.1723

AVR, auditory evoked visual cortex responses induced by auditory stimulus on both ears; LSLV, left auditory stimulation evoked left visual cortex responses; LSRV, left auditory stimulation evoked right visual cortex responses; RSLV, right auditory stimulation evoked left visual cortex responses; RSRV, right auditory stimulation evoked right visual cortex responses.

a *p *<* *.05.

**Table 4:** Comparison of bilateral AVR between monocularly blind and normal rats

	Frequency	Monocularly blind rats	Normal rats	*p* value
AVR	8000	41 ± 2	30 ± 1	.00204^a^
	16,000	58 ± 2	53 ± 2	.14049
	24,000	63 ± 2	61 ± 2	.69966
	32,000	77 ± 2	76 ± 2	1.00000

AVR, auditory evoked visual cortex responses induced by auditory stimulus on both ears.

a *p *<* *.05.

**Figure 8:**
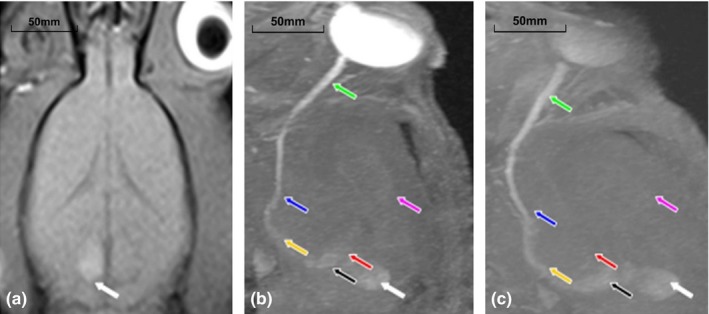
(a) Hyperintensity in the left superior colliculus shown by MEMRI 4 months after monocular blindness; (b) Left side of visual pathway shown by MEMRI (TSMIP) 4 months after monocular blindness; (c) Left side of the visual pathway shown by MEMRI (TSMIP) 4 months after monocular blindness. Note: optic nerve (green arrow), optic chiasm (blue arrow), optic tract (yellow arrow), lateral geniculate nucleus (black arrow), superior colliculus (white arrow), optic radiation (red arrow), visual cortex (purple arrow)

The SI of the right SC in all monocularly blind rats increased on T1WI on MEMRI, whereas the SI of the left SC slightly increased, indicating successful establishment of the monocularly blind rat model 4 months after right ON transection. T1WI 3D FLASH imaging was further performed, and all the images were processed after three‐dimensional reconstructions. TSMIP can stereoscopically (3D) show the entire visual pathway in monocularly blind rats (Figure 8), while MPR and MIP can only demonstrate some structures of visual pathway clearly simultaneously.

**Figure 9:**
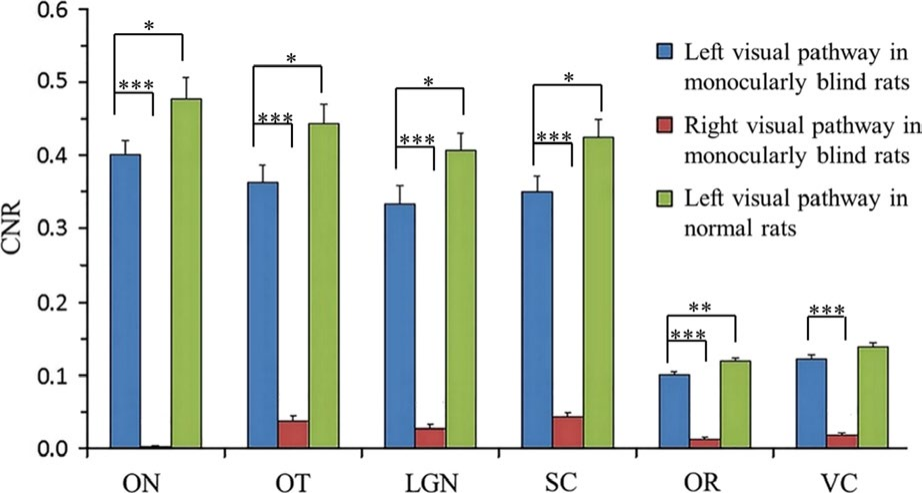
Comparison of the CNR of the visual pathway between the left and the right side in monocularly blind rats and between normal and monocularly blind rats on the left side. ON, optic nerve; OT, optic tract; LGN, lateral geniculate nucleus; SC, superior colliculus; OR, optic radiation; VC, visual cortex. ***: *p *<* *.001, **: *p *<* *.01, *: *p *<* *.05

**Figure 10:**
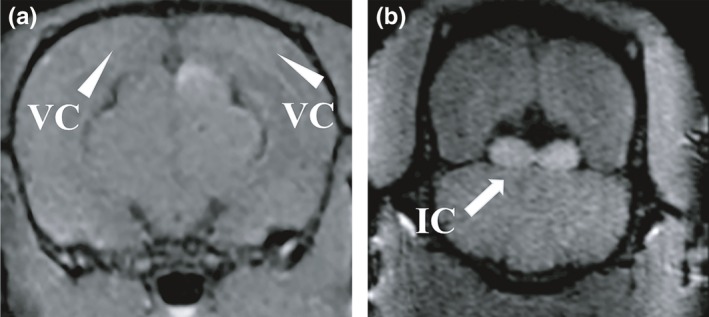
(a) TSMIP showed the signal intensity of the left visual cortex (the blind eye side) slightly increased and were approximate to the signal intensity of the right visual cortex (the remaining eye side); (b) TSMIP showed high signal intensity in bilateral inferior colliculi

In the monocularly blind rats, the CNR of the representative structures (including ON, OT, LGN, SC, OR and VC) of visual pathway projected from the left eye were significantly higher than those of visual pathway projected from the right eye (*p *<* *.001). Moreover, there existed a slight difference in the CNR of the visual pathway between the non‐blind eyes (left eyes) of the monocular blind rats and left eyes of the normal rats. Specifically, the CNR of each representative structure in the visual pathway projected from the left eye of monocular blind rats was significantly lower than those of normal rats (*p *<* *.05) (Table 5 and Figure 9). Interestingly, we found that the SI of the left VC and the bilateral inferior colliculi (IC) in one rat of Group A were slightly enhanced (Figure 10).

**Table 5:** Comparison of CNR of visual pathway between the left and right side in monocularly blind rats and between monocularly blind rats and normal rats in the left side

Site	Monocularly blind rats		Normal rats	*p* value	
	Left pathway	Right pathway	Left pathway	LRMB	LMLN
ON	0.4002 ± 0.0204	0.0034 ± 0.0007	0.4773 ± 0.0289	.0000***	.0499*
OT	0.3624 ± 0.0238	0.0375 ± 0.0071	0.4429 ± 0.0262	.0000***	.0419*
LGN	0.3336 ± 0.0238	0.0276 ± 0.0053	0.4072 ± 0.0231	.0000***	.0479*
SC	0.3500 ± 0.0219	0.0432 ± 0.0058	0.4251 ± 0.0236	.0000***	.0380*
OR	0.1010 ± 0.0045	0.0126 ± 0.0025	0.1203 ± 0.0033	.0000***	.0045*
VC	0.1228 ± 0.0052	0.0184 ± 0.0037	0.1388 ± 0.0054	.0000***	.0536

ON, optic nerve; OT, optic tract; LGN, lateral geniculate nucleus; SC, superior colliculus; OR, optic radiation; VC, visual cortex. LRMB, comparison of CNR of visual pathway between the left and right side in monocularly blind rats; LMLN, comparison of CNR of visual pathway between monocularly blind rats and normal rats in the left side.

***p <* *.001, *p <* *.05.

The AVR results indicated that the corresponding VC of the blind and non‐blind eyes in monocularly blind rats did not respond to an auditory stimulus (dissimilation or compensatory pathway) or only displayed dissimilation under a low‐frequency auditory stimulus, which was in accordance with the result of MEMRI with the slightly enhanced SI of bilateral ICs.

## 4 DISCUSSION

There is an increasing number of studies on the changes in the visual cortex caused by monocular visual plasticity that use advanced technologies such as BOLD‐fMRI, VEP, and HRP (Chow et al., 2011; Dietrich et al., 2015; Jeffery & Thompson, 1986; Mastropasqua et al., 2015; Qu et al., 1996; Toldi et al., 1996). However, the above research methods have obvious limitations and shortcomings, such as poor spatial resolution, a certain degree of subjectivity, restriction to particular regions of the visual cortex, and incomplete or unclear demonstration of neural visual pathways. Manganese‐enhanced MRI (MEMRI) is a rapidly developing technique that was introduced by Lin and Koretsky and has been applied to extensively research the neural pathways in vivo in animals (Watanabe, Frahm, & Michaelis, 2004). In contrast to other research methods mentioned above concerning neural pathways or cerebral function, MEMRI can specifically, dynamically and directly track neural conduction pathways in vivo and has other advantages, including simplicity, high spatial resolution, strong visibility, independence from the change of hemodynamics, the ability to precisely measurement the volumes of minute structures in the brain, and the ability to observe the activity and localization of each encephalic region (Berkowitz, Roberts, Goebel, & Luan, 2006; Drapeau & Nachshen, 1984; Geraldes, Sherry, Brown, & Koenig, 1986; Hu, Pautler, MacGowan, & Koretsky, 2001; Hunter, Komai, Haworth, Jackson, & Berkoff, 1980; Narita, Kawasaki, & Kita, 1990; Pautler & Koretsky, 2002). Although MEMRI had been utilized by Chan et al. (2012) to study the changes in bilateral visual pathways in monocularly blind animals, currently, no systematic studies have been reported in which MEMRI combined with electrophysiology tests (VEP and AVR) was used to explore the neural plasticity of the visual pathway in monocularly blind models. Thus, the purpose of our study was to investigate the changes in the visual pathway after monocular deprivation using MEMRI and to verify them using VEP and AVR.

In this present study, we found that the VEP in the left VC were smaller than those in the right. However, there were no significant differences, which was probably due to the neural plasticity of the VC or a small sample size in our study. In theory, ON transection may lead to the disappearance of VEP signals (Toldi et al., 1996). However, measured VEP have spontaneous waveforms that are generated by residual signals in the VC and other regions of the cerebral cortex, which is also called the background waveform (baseline VEP). For the same monocularly blind rats, the amplitude of the VEP after ON transection was significantly lower than before transection (*p *<* *.001) and was almost equal to the baseline VEP, with no significant difference. Consequently, VEP recorded in rats with ON transection were not specific visual signals but were actually the residual signal, which confirmed the complete visual loss caused by ON transection. In normal rodents, only approximately 5% to 10% of visual signals arrive at the ipsilateral visual cortex (Chan et al., 2012). Hence, unilateral optic nerve transection should lead to approximately 5% to 10% loss of VEP signals (recorded in the ipsilateral visual cortex) in the contralateral eye without ON transection. However, in our study, the loss of VEP signals was much >10% in both the blind eyes and the non‐blind eyes. The possible reasons may be as follows: (1) the visual pathway and VC had degenerated by 4 months after unilateral ON transection, which often simultaneously happens in bilateral visual pathways; (2) the crosslinking and interaction between bilateral VCs (via the integrated corpus callosum) intensified the decreased degree of VEP amplitude in the non‐blind eyes (Toldi et al., 1996).

In previous studies, excitement and dissimilation can be demonstrated in the VC of binocularly blind animals using AVR (Bronchti, Heil, Scheich, & Wollberg, 1989; Bronchti et al., 2002; Izraeli et al., 2002). However, to our knowledge, the study using AVR to determine the dissimilation in the VC of unilaterally blind rats has not been reported. Our study was inspired by previous AVR studies of binocular blindness and investigated the responses of bilateral VCs before and after monocular blindness using AVR. The data from the four groups of VC responses to bilateral auditory stimuli (LSLV, LSRV, RSLV and RSRV) were recorded to observe whether there were nerve fiber connections between the VC and the auditory pathway, and the four curves were generally similar. Although there was a significant difference at the low frequency, which was probably caused by noise, we found no significant differences overall. The results revealed that the corresponding VCs of the blind eye and the non‐blind eye did not cause significant dissimilation. Specifically, the bilateral VCs had no response to the auditory stimulus or only responded to the low frequency, which indicated that the auditory compensatory pathway was most likely established at the low frequency. However, our experimental results still need to be validated in a larger sample size in the further research.

Consistent with the results in the study by Chan et al. (2012), the right SC on T1WI in monocularly blind rats showed hyperintensity, whereas the left SC only showed slight signal enhancement, suggesting successful establishment of monocularly blind rat models after 4 months of monocular deprivation. The LGN and SC, which were shown in the study by Chan et al. (2012), as well as other structures in the visual pathway (including OT, OR and OC), were demonstrated on TSMIP after tridimensionally reconstructing T1WI 3D FLASH imaging. Additionally, the CNR of the six representative structures of the visual pathway projected from the left eye seen on MEMRI were significantly higher than those of the visual pathway projected from the right eye. In other words, the SI of the visual pathway projected from the left eye was significantly higher than that of the visual pathway projected from the right eye. Moreover, due to the administration of higher doses of MnCl_2_ and TSMIP in this study than in Chan et al. (2012), we found that an additional three structures had similar manifestations as in the study by Chan et al. Our results suggest that the optimal injection doses or reconstruction methods play an important role in the signal change of the entire visual pathway in MEMRI.

Although there were no significant differences between bilateral VEPs, the findings of the MEMRI were similar to the results of the VEP test, which showed that the VEP of the left VC was smaller than that of the right VC. The possible reasons were complicated and included baseline VEP (background), the plasticity of the visual cortex, the small sample size, disturbance from anesthesia, and the interaction and influence between bilateral VCs via the corpus callosum (Chan et al., 2012; Toldi et al., 1996). Hence, further studies should be performed with larger sample sizes and controls for other influencing factors. In humans and rats, each eye projects bilaterally, with the left visual field (right retina) projecting to the right visual cortex. The visual field in humans, and less so in rats, is largely binocular, so most neurons in the cortex receive input from both eyes. In our study, MEMRI results suggested the existence of a visual compensation pathway in the non‐blind eye after monocular blindness, whereas the VEP signals of the non‐blind eye also significantly decreased due to the decrease and degeneration of visual pathway function in the non‐blind eye caused by the interconnection via the corpus callosum, which prompted simultaneous functional compensation and functional degeneration (Toldi et al., 1996).

Moreover, the CNR of each representative structure in the visual pathway projected from the left eye of monocularly blind rats was significantly lower than those of normal rats (*p *<* *.05), which was in accordance with the VEP results in normal rats and monocularly blind rats (*p *<* *.001). The SI of the left VC (the blind eye side) of one rat was found to be increased, which was consistent with the results of Toldi et al. (1996), who detected induced responses in bilateral primary and secondary lateral VCs using electrophysiology by stimulating the corpus callosum. Furthermore, Chan et al. (2014) found that loss of visual input after monocular blindness may not affect the uptake and anterograde monosynaptic transport of Mn^2+^ in the interhemispheric connections (the corpus callosum), suggesting that after monocular blindness the corpus callosum is still capable of transporting Mn^2+^ to the contralateral hemisphere. Moreover, the left VC is most nearest structure to the corpus callosum, and therefore it can be slightly enhanced after intravitreal injection of MnCl_2_. Interestingly, we also found that the SI of the bilateral ICs in one rat was slightly increased, which was probably due to connections among the IC, LGN and SC, allowing MnCl_2_ to reach the IC via the bilateral LGNs or SCs (Chuang & Koretsky, 2009; Doron & Wollberg, 1994; Skaliora, Doubell, Holmes, Nodal, & King, 2004; Toldi et al., 1996; Westby, Keay, Redgrave, Dean, & Bannister, 1990). However, the existence of the auditory compensation pathway at the level of the IC still needs to be validated in further studies. Overall, MEMRI did not show distinct nerve fiber connections between the visual cortex and auditory cortex, which was in agreement with the results of AVR in our study.

Our study has some limitations. First, since the procedure of right superior oblique muscle transection may affect eye movements of infant rats, sham‐operated animals for a control group should have been adopted in the further study. Second, although MEMRI combined with VEP and AVR helped to verify a previous report that revealed the reorganization of various structures in the visual pathway of neonatal monocularly blind rats, it remains unclear if such excitability and heterization occurred in the first (V1) and the second (V2) visual cortex at the same time. The in‐depth study of various functional regions of the visual cortex, such as BA17, 18, 19 and 37, has not been performed. The relationships among these changes at various time points (early, medium, and late) within the critical period after monocular deprivation of newborn rats are still unknown. Third, in monocularly blind and normal rats, although the ICs can transmit information to LGN and SC, respectively (Chuang & Koretsky, 2009; Doron & Wollberg, 1994; Skaliora et al., 2004; Toldi et al., 1996; Westby et al., 1990), we have no idea if the information can be transmitted from SC upward to the visual cortex in the same way as the LGN. In other words, except for the LGN, it is unknown if bilateral auditory compensatory pathways are established via the SC. Finally, the sample size in this study was still too small. Additionally, our study was performed on a 3.0‐T MRI scanner with a moderate spatial resolution, resulting in the SI differences between left and right side difficult to determine. Thus, a larger sample size and high tesla (such as 9.4T or 7T) MR scanner should be adopted in our further study.

In conclusion, the results of the MEMRI were verified by VEP and AVR, indicating that MEMRI combined with electrophysiology may be potentially helpful in the evaluation of the possible generation of new visual/auditory compensatory pathways after monocular blindness.

## STATEMENT FOR AVAILABILITY OF DATA AND MATERIALS

Due to statutory provisions regarding data‐ and privacy protection, the dataset supporting the conclusions of this article is available upon individual request directed to the corresponding author.

## CONFLICT OF INTERESTS

None declared.
